# Clarified Açaí (*Euterpe oleracea*) Juice as an Anticonvulsant Agent*: In Vitro* Mechanistic Study of GABAergic Targets

**DOI:** 10.1155/2018/2678089

**Published:** 2018-03-20

**Authors:** Gabriela P. F. Arrifano, Mathieu P. Lichtenstein, José Rogério Souza-Monteiro, Marcelo Farina, Hervé Rogez, José Carlos Tavares Carvalho, Cristina Suñol, Maria Elena Crespo-López

**Affiliations:** ^1^Departament of Neurochemistry and Neuropharmacology, Institut d'Investigacions Biomèdiques de Barcelona (IIBB), Consejo Superior de Investigaciones Científicas (CSIC), IDIBAPS, CIBERESP, Barcelona, Spain; ^2^Laboratório de Farmacologia Molecular, Instituto de Ciências Biológicas, Universidade Federal do Pará, Belém, PA, Brazil; ^3^Departamento de Bioquímica, Centro de Ciências Biológicas, Universidade Federal de Santa Catarina, Florianópolis, SC, Brazil; ^4^College of Food Engineering and Centre for the Valorization of Bioactive Compounds from Amazonia, Universidade Federal do Pará, Belém, PA, Brazil; ^5^Laboratório de Pesquisa em Fármacos, Departamento de Ciências Biológicas e da Saúde, Universidade Federal do Amapá, Macapá, AP, Brazil

## Abstract

Seizures affect about 50 million people around the world. Approximately 30% of seizures are refractory to the current pharmacological arsenal, so, the pursuit of new therapeutic alternatives is essential. Clarified *Euterpe oleracea* (EO) juice showed anticonvulsant properties similar to diazepam in an *in vivo* model with pentylenetetrazol, a GABA_A_ receptor blocker. This study investigated the effects of EO on the main GABAergic targets for anticonvulsant drugs, analyzing the effect on the GABA receptor's benzodiazepine and picrotoxinin binding sites and the GABA uptake. Primary cultures of cortical neurons and astrocytes were treated with EO (0–25%) for up to 90 min. [^3^H]Flunitrazepam and [^3^H]TBOB binding, [^3^H]GABA uptake, cell viability, and morphology were assayed. Nonlethal concentrations of EO increased agonist binding and decreased antagonist binding in cortical neurons. Low concentrations significantly inhibited GABA uptake, especially in astrocytes, suggesting an accumulation of endogenous GABA in the synaptic cleft. The results demonstrate, for the first time, that EO can improve GABAergic neurotransmission via interactions with GABA_A_ receptor and modulation of GABA uptake. Understanding these molecular mechanisms will help in the treatment of seizures and epilepsy, especially in developing countries where geographic isolation and low purchasing power are the main barriers to access to adequate treatment.

## 1. Introduction

Seizures are deleterious consequences of serious insults to the brain (e.g., trauma and stroke) and primary manifestations in epilepsy, affecting more than 50 million people worldwide [1]. Approximately 30% of seizures are refractory to the current pharmacological arsenal. Nearly 80% of all epileptic patients live in low-income or developing countries, and approximately 75% of these patients do not get adequate treatment [1]. Geographic isolation and low purchasing power are the main barriers to access the treatment in these countries, so, an easily available fruit juice may have an important impact.

Recently, the potent anticonvulsant properties of açaí (*Euterpe oleracea* Martius, family Arecaceae) were demonstrated in an *in vivo* pentylenetetrazol (PTZ) mouse model [2]. Açaí is the fruit of a common palm found in the eastern Amazonian floodplains, and its juice is highly consumed in northern Brazil (up to 1 l/day per person) [3]. It is also available at the international market as a growing economic value (e.g., in 2011, açaí generated an estimated monetary movement of US$ 700,000 in Brazil) [4].

In the previous study, four doses of clarified açaí juice (10 *μ*l/g body weight, equivalent to approximately 700 ml/day for a person weighing 70 kg) were sufficient to significantly protect against PTZ-induced seizures and seizure-related oxidative stress in mice [2]. Understanding the molecular mechanisms underlying this effect will help in the treatment of the disease. Considering that PTZ blocks the chloride channel coupled to the GABA_A_ receptor complex, the present study aimed to analyze the possible modulation of GABAergic homeostasis within synaptic clefts *in vitro*.

## 2. Material and Methods

### 2.1. Culture of Neurons and Astrocytes

Animals were handled in compliance with protocols approved by the Autonomous Government of Catalonia, Spain, following European Union guidelines. All efforts were carried out to reduce the number of animals and minimize their suffering.

Primary cultures of neocortical neurons were obtained from 16-day-old NMRI mouse embryos (Charles River, Iffa Credo, Saint-Germain-sur-l'Arbresle, France) [5, 6]. The cell suspension for astrocyte culture was obtained in the same way as for neurons. Cells were grown for 2 weeks in DMEM : F12 containing 10% fetal bovine serum (FBS) until reaching confluence. Cytosine arabinoside (AraC) 10 *μ*M was added to the media the last 2 days to prevent proliferation of other glial cells. Cultures were harvested with trypsin-EDTA, gently disaggregated, and seeded in 24-well plates at a density of 450,000 cells/ml with DMEM : F12 plus 10% FBS for 15 days. Thereafter, 200 *μ*M dibutyryl cAMP was added to fully differentiate the astrocytes.

### 2.2. Clarified *Euterpe oleracea* (EO) Juice

Amazon Dreams (Belém, Pará, Brazil) kindly provided the commercial clarified *Euterpe oleracea* Martius, family Arecaceae, juice used in this work. The patented process to produce the juice was licensed by both Amazon Dreams and Universidade Federal do Pará (PI 8 1003060-3). It includes the microfiltration and centrifugation of a juice prepared with fresh fruit [2]. In order to quantify the anthocyanins and major flavonoids present in clarified juice, two validated UHPLC-DAD methods were used [7, 8], with the standard compounds (orientin, homoorientin, taxifolin, cyanidin 3-glucoside, and cyanidin 3-rutinoside) purchased from Extrasynthèse.

### 2.3. Treatments

After 7–12 days *in vitro* (div), the culture medium was removed and cells were rinsed with Hank's buffer. Cells were treated with 0–25% EO in Hank's buffer (250 *μ*l final volume) for binding and uptake assays. Osmolality was maintained at 257–332 mOsmol/kg (data not shown) as recommended [9]. Cell viability and morphology were evaluated after exposure to 0–50% EO.

### 2.4. [^3^H]Flunitrazepam Binding

After treatment with EO for 30 min, binding to the benzodiazepine site of the GABA_A_ receptor in neuronal cultures was assayed using 1.83 nM [^3^H]flunitrazepam according to Sunol et al. [6] and Garcia et al. [5]. [^3^H]Flunitrazepam (specific radioactivity 82.5 Ci/mmol) was purchased from Amersham, Life Sciences. Data were expressed as the percentage of basal specific binding.

### 2.5. [^3^H]-t-Butylbicycloorthobenzoate ([^3^H]TBOB) Binding

Cultured cortical neurons were treated with EO for 60 min and binding to the picrotoxinin site at the GABA_A_ receptor channel assayed using 1.84 nM [^3^H]TBOB in Hank's buffer. [^3^H]TBOB (16.2 Ci/mmol) was purchased from Amersham, Life Sciences. The method in van Rijn et al. [10] was adapted to evaluate TBOB binding in intact cells (unpublished results). Nonspecific binding was determined in the presence of 100 *μ*M picrotoxinin. After 30 min of incubation at 25°C, cold buffer was added and rapidly removed by suction. The cells were rinsed two times with cold buffer and disaggregated with 0.2 N NaOH overnight at 4°C. The radioactivity of the samples was quantified by liquid scintillation spectroscopy using the OptiPhase cocktail (Wallac, UK). Data were expressed as the percentage of basal specific binding.

### 2.6. [^3^H]GABA Uptake

After 55 min of treatment with EO, [^3^H]GABA uptake was assayed in both mature cultures of neurons and astrocytes using 1.46 nM [^3^H]GABA according to Vale et al. [11]. [^3^H]GABA (90 Ci/mmol) was purchased from Amersham, Life Sciences. Data were expressed as the percentage relative to the control group.

### 2.7. Cell Viability and Morphology

After 90 min of treatment with EO, cell morphology and cytoskeletal performance was determined by tau immunocytochemistry (primary antibody, Sigma T-6402, 1 : 1000) as described elsewhere [12]. The cell viability of neuronal cells was evaluated using the 4,5-dimethylthiazol-3,5-diphenyltetrazolium (MTT) method as described previously [13]. Cell viability was reported as the percentage of reduced MTT compared to the control group.

### 2.8. Data Analysis

The results are presented as mean ± SEM of at least three independent experiments performed in triplicate. Student's *t*-test and one-way analysis of variance (ANOVA) followed by Dunnett's post hoc test were applied. Significance was set at *P* < 0.05.

## 3. Results

EO showed five major phenolic compounds, all expressed per 100 ml of juice: 38 mg of orientin, 25 mg homoorientin, 31 mg of taxifolin deoxyhexose, 18 mg of cyanidin 3-glucoside, and 45 mg of cyanidin 3-rutinoside.

**Figure 1:**
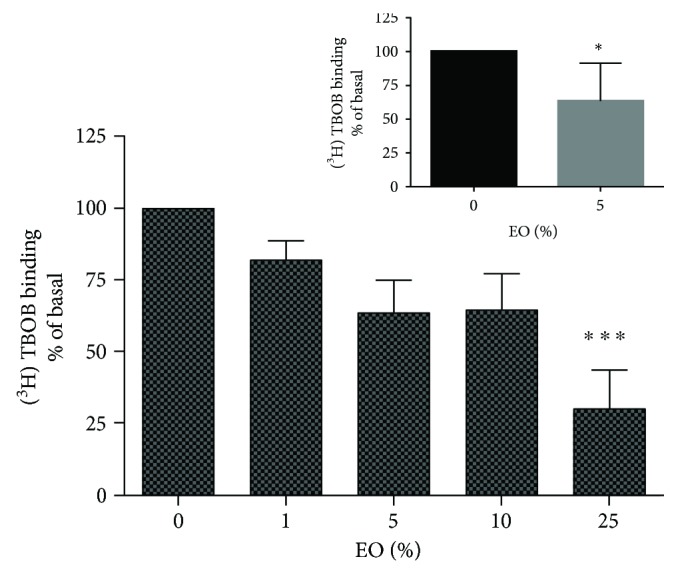
[^3^H]TBOB binding in cultures of cortical neurons treated with clarified *Euterpe oleracea* (EO) juice. All data were evaluated by one-way ANOVA followed by the Dunnett' post hoc test, except data in inset that were evaluated using *t*-test. ^∗^*P* < 0.05 and ^∗∗∗^*P* < 0.001 versus control.

Treatment with EO significantly decreased [^3^H]TBOB binding of the GABA_A_ receptor in cortical neurons treated with ≥25% EO (Figure 1). Because of a high number of groups and the use of demanding statistical tests (such as ANOVA followed by Dunnett) could ignore slight differences between groups, an additional analysis with *t*-test was also carried out between control and EO-treated groups. In this analysis, 5% EO was sufficient to significantly reduce [^3^H]TBOB binding (Figure 1, inset).

**Figure 2:**
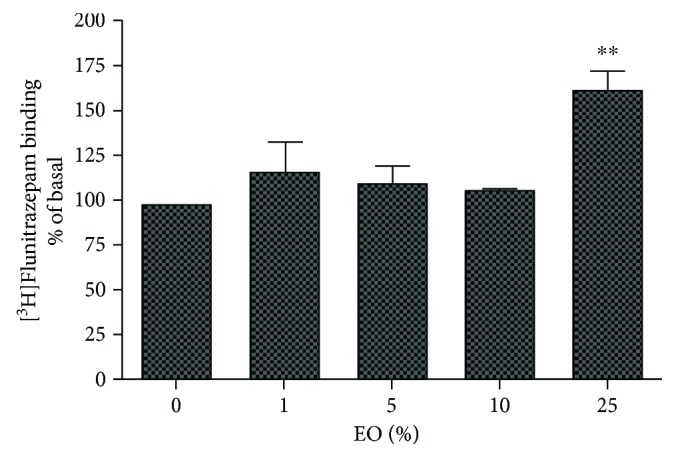
[^3^H]Flunitrazepam binding in cultures of cortical neurons treated with clarified *Euterpe oleracea* (EO) juice. Data were evaluated by one-way ANOVA followed by the Dunnett's post hoc test. ^∗∗^*P* < 0.01 versus control.

A significant increase in [^3^H]flunitrazepam binding of the GABA_A_ receptor (>50%) was observed after treatment with 25% EO (Figure 2).

**Figure 3:**
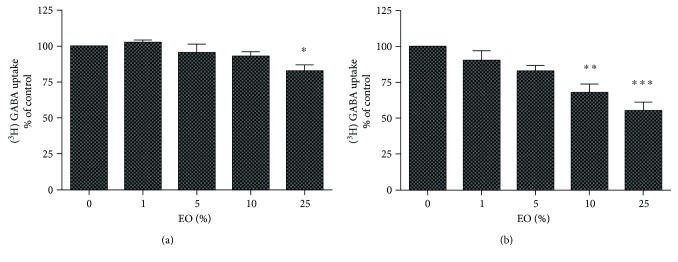
[^3^H]GABA uptake in cultures of cortical neurons (a) and astrocytes (b) treated with clarified *Euterpe oleracea* (EO) juice. Data were evaluated by one-way ANOVA followed by the Dunnett's post hoc test. ^∗^*P* < 0.05, ^∗∗^*P* < 0.01 and ^∗∗∗^*P* < 0.001 versus control.

Treatment with 25% EO significantly inhibited the [^3^H]GABA uptake in cortical neurons (Figure 3(a)). Interestingly, cultures of cortical astrocytes were more sensible to the effect of EO on [^3^H]GABA uptake, showing significant dose-response inhibition with ≥5% EO (Figure 3(b)).

**Figure 4:**
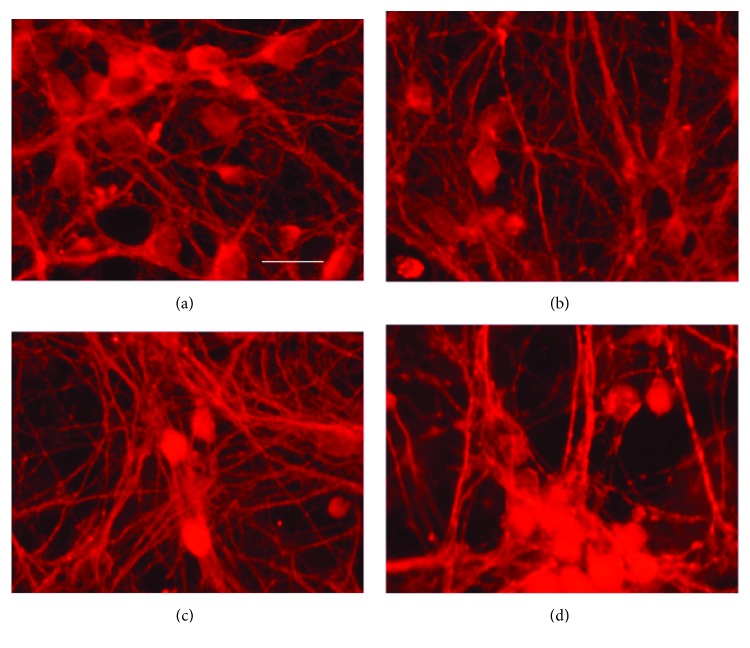
Representative micrographs of cortical neurons exposed to clarified *Euterpe oleracea* (EO) juice for 90 minutes. (a) Control; (b) 10% EO; (c) 25% EO; and (d) 50% EO. Neurons were labelled with anti-tau, allowing a comparison of neuronal arborization among treatments. Scale bar = 20 *μ*m.

**Figure 5:**
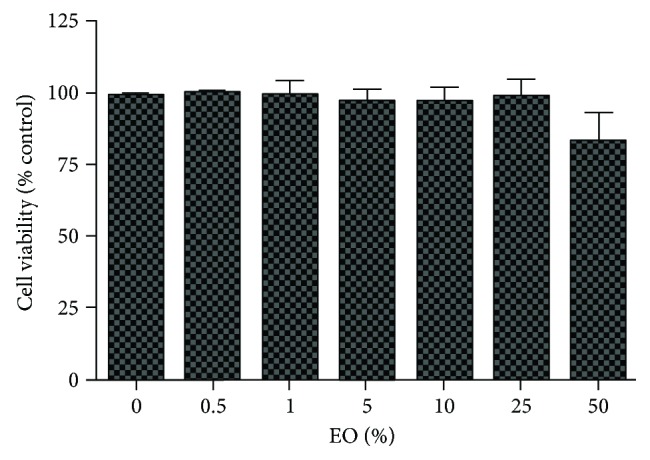
Cell viability of cortical neurons exposed to clarified *Euterpe oleracea* (EO) juice for 90 minutes. No significant difference was found between groups using one-way ANOVA.

Exposure to EO (0–50%) did not alter the cell morphology (Figure 4) or reduce cell viability (Figure 5).

## 4. Discussion

This work demonstrated, for the first time, that EO is able to interact with GABA_A_ receptor and affect GABA uptake. Noncytotoxic concentrations of EO increased flunitrazepam binding and decreased TBOB binding in cortical neurons. Lower concentrations of EO significantly inhibited GABA uptake, especially in astrocytes. These events could possibly lead to the accumulation of endogenous GABA in the synaptic cleft and enhanced inhibitory neurotransmission in the brain.

Here, we used primary cultures of cortical neurons and astrocytes. After 7-8 days *in vitro*, neocortical neurons are mature and comprise mainly of GABAergic neurons [11], making them an excellent model for mechanistic studies. In these cells, GABA_A_ receptor is a major pharmacological target for anticonvulsant drugs, such as benzodiazepines.

Although the allosteric modulation of GABA_A_ receptor via multiple drug-binding sites is very complex (reviewed by [14]), the main targets for flunitrazepam and TBOB on the channel are the anticonvulsant benzodiazepine site and the convulsant picrotoxinin site, respectively. Thus, analysis of the interaction between the GABA receptor and possible therapeutic candidates using radioligand-binding assays is well recognized for elucidating the molecular mechanisms underlying the effect of anticonvulsant/proconvulsant agents [5, 15, 16]. Positive allosteric modulators or agonists of GABA_A_ receptor exhibiting anticonvulsant actions can increase the binding of [^3^H]flunitrazepam and/or modify the binding of [^3^H]TBPS, a TBOB analog [5]. For example, the anticonvulsant drug felbamate inhibits [^3^H]TBOB binding and increases chloride current (an indicative of a possible opening of the channel) [15]. Our results demonstrate that exposure to EO positively modulates the benzodiazepine site in addition to the possibly more potent negative modulation of the picrotoxinin site (Figures 1 and 2). Both actions facilitate the inhibitory role of GABA in the brain, making the initiation and propagation of exacerbated excitatory activity, as occurs in a seizure, more difficult.

Other important molecular targets for anticonvulsant drugs are the GABA transporters (GATs), which are inhibited by the anticonvulsant tiagabine [16, 17]. EO significantly inhibited GABA uptake in both cortical neurons and astrocytes (Figure 3). EO was particularly potent in astrocytes, causing significant inhibition at less than half the concentration than in neurons (Figure 3). Hypothetically, this finding may point to a more potent effect of EO toward GAT3, which is mainly found in astrocytes, than GAT1, which is mainly found in neurons. Interestingly, the inhibition of astrocytic GAT could have a superior anticonvulsant effect because GABA is nonreutilized and effectively eliminated in astrocytes, leading to clearance of this neurotransmitter from presynaptic neurons [17]. Inhibition of GABA reuptake has been shown to have significant therapeutic efficacy in other models [18], indicating a possible beneficial effect of EO in comorbidities such as depression and anxiety. By blocking GAT, endogenous GABA accumulates in the synaptic cleft, increasing inhibitory neurotransmission. This high levels of GABA caused by incubation with EO could be responsible, partially at least, for the effects detected in the receptor, since GABA is able of increasing benzodiazepine binding and decreasing TBOB binding. Additionally, the possible presence of GABA in the composition of EO must not be discarded, since this neurotransmitter is a constituent found in many plants [19]. However, to date, GABA has not been described as a main component of EO and it seems to be unprovable that the anticonvulsant effect of EO can be totally attributed to this compound due to the absence of sedative effects of EO [2].

Moreover, our data showed that doses of EO that affect the GABA receptor and uptake did not alter cellular viability or morphology (Figures 4 and 5), confirming that they are not the consequence of a reduced number of cells and suggesting that EO acts on diverse molecular targets in the GABAergic system. The latter hypothesis is in agreement with the notable potency of the anticonvulsant effect of EO observed *in vivo* [2]. In a similar model of PTZ-induced seizures, diazepam (3 mg/kg) reduced the duration of clonic-tonic convulsions in a similar way as EO (9.2 ± 1.5 and 8.0 ± 1.12 seconds for diazepam and EO, resp.; *P* > 0.05, unpaired *t*-test). Also, EO caused a higher delay of the onset (405.1 ± 99.71 seconds) than that of diazepam (187 ± 7.2 seconds; *P* < 0.001, unpaired *t*-test) [2, 20]. Although our initial hypothesis was that the anticonvulsant effect of EO is due to its extraordinary antioxidant properties because a 1 : 100 EO dilution had greater scavenger action than 800 *μ*M Trolox [2], this study is the first to show that EO also significantly influences on the GABAergic system.

Commercial EO was used to guarantee that the samples were indicative of human consumption. All macronutrients (i.e., lipid, fiber, and protein) are eliminated in the clarification process, reducing possible interference by these compounds. So, phenolic compounds, particularly anthocyanins, are the main compounds in this juice. Preliminary analysis of the samples indicated 1662.15 mg gallic acid equivalents/l of phenolic compounds, including 761 mg cyanide equivalents/l of anthocyanins. Major flavonoids present in clarified açaí were (per 100 mL) cyanidin 3-rutinoside (45 mg), orientin (38 mg), taxifolin deoxyhexose (31 mg), homoorientin (25 mg), and cyanidin 3-glucoside (18 mg). Many flavonoids are able to interact with the benzodiazepine site of GABA receptors and modulate the chloride flux [16, 19]. Interestingly, some of these flavonoids exhibit anticonvulsant activity in the absence of sedative effects [16]. Although additional studies are necessary, this finding may explain the effect of EO, found by Souza-Monteiro et al. and this work, which modulates the GABA receptor without sedative effects [2].

Our results demonstrate that EO can improve GABAergic neurotransmission via interactions with GABA_A_ receptor and modulation of GABA uptake. These events could possibly lead to the accumulation of endogenous GABA in the synaptic cleft and enhanced inhibitory neurotransmission in the brain.

Knowledge of the molecular mechanisms underlying the anticonvulsant effect of açaí is of particular importance for use in folk medicine by isolated populations. Many of these populations live in the Amazon, where compliance with chronic pharmacological treatment with current anticonvulsant drugs is reduced due to socioeconomic factors (i.e., poverty and isolation) that make it difficult to access health services. Thus, a common palm widely distributed in the Amazon could be an extremely useful tool for treating seizures, especially in these populations.
